# Improving Knowledge and Attitudes towards Depression: a controlled trial among Chinese medical students

**DOI:** 10.1186/1471-244X-11-36

**Published:** 2011-03-08

**Authors:** Ye Rong, Nick Glozier, Georgina M Luscombe, Tracey A Davenport, Yueqin Huang, Ian B Hickie

**Affiliations:** 1 Brain & Mind Research Institute The University of Sydney, Sydney, Australia Level 4, 94 Mallett Street Camperdown NSW 2050, Australia; 2 Disciplines of Psychiatry and Sleep Medicine The University of Sydney, Sydney, Australia Level 4, 94 Mallett Street Camperdown NSW 2050, Australia; 3 Academic Research and Statistical Consulting (ARSC) 5 Herbert Street, West Ryde NSW 2114, Australia; 4 Institute of Mental Health Peking University, Beijing, China No.51 Hua Yuan Bei Road, Haidian District, Beijing PR China 100083

## Abstract

**Background:**

Establishing an evidence-based method of improving knowledge and attitudes concerning depression has been identified as a priority in Chinese medical education. The purpose of this study was to determine whether a self-directed learning strategy as a part of student-centred education improved knowledge of and attitudes towards depression among Chinese medical students.

**Methods:**

A controlled trial in which 205 medical students were allocated to one of two groups: didactic teaching (DT) group or a combined didactic teaching and self-directed learning (DT/SDL) group. The DT/SDL group continued having a series of learning activities after both groups had a lecture on depression together. Student's knowledge and attitudes were assessed immediately after the activities, one month and six months later.

**Results:**

The intervention (DT/SDL) group showed substantially greater improvements in recognition of depression as a major health issue and identifying helpful treatments than the DT group. Only the DT/SDL group demonstrated any improvement in attitudes. This improvement was sustained over six months.

**Conclusions:**

Self-directed learning is an effective education strategy in improving medical students' knowledge of and attitudes towards depression.

## Background

Depression is one of the leading causes of premature death or lifetime disability in China [1]. As in many other countries, recognition and treatment of depressive disorders remains problematic [2-4]. The range of reasons that contribute to under diagnosis and inadequate treatment include professional related factors such as lack of detailed knowledge of the condition, lack of confidence in available treatments, demands on consultation time and stigma [5,6]. While the public health aspects of psychiatry are largely neglected in medical education worldwide [7], we have previously reported that the public health impact of depression is more widely known among Australian than Chinese medical students [8]. Other issues related to under-treatment of mental disorders in China include immense population size, inadequacies of the health system including poor mental health expenditure, lack of mental health specialists especially in rural areas, as well as stigma in both the community and among health professionals [9,10]. A lack of medical or personal knowledge of common mental health problems like depression may contribute to negative attitudes and reinforce health services neglect and other discriminatory behaviour [11,12].

Until recently almost all highly-trained doctors in China worked in hospitals and predominantly in specialist clinics. Medical care in China is now in transition with a small, but increasing, number of doctors working in community health centres and providing more general care [13]. Thus the vast majority of medical presentations of depression will be to doctors without specific mental health expertise. However, only approximately 8% of people suffering from a mood disorder will make contact with a treatment provider for their condition in China [9]. Thus there will be a growing reliance in China upon non-specialist doctors to achieve early recognition and treatment. Providing adequate training in common mental disorders for health professionals is now a priority for the Chinese government [14]. As such, specific interventions to increase knowledge and reduce negative attitudes among Chinese medical students are particularly timely.

Previous studies have addressed the impact of didactic interventions to improve knowledge and negative attitudes associated with mental illness in medical students [15-17]. Mino's study addressed the stigma towards "mental illness" with a one-hour lecture and, like the others, showed some positive short term effects, predominantly on social distance [18]. Although the traditional "teacher-centred" style teaching has continued in most medical schools in China, some leading medical schools have been carrying out a series of medical education innovations and are adopting a "student-centred" education style [19]. To our knowledge, the use of such an education style in psychiatry has not been evaluated in this context. The aim of this controlled trial was to determine whether a context specific, student-centred educational intervention increased the knowledge of depression and improved attitudes towards depression among Chinese medical students and to evaluate how sustained was any change.

## Methods

### Setting, participants and allocation

The study was conducted at the Health Science Centre of Peking University, China. Medical students at this university are selected through National Higher Education Entrance Examination (NHEEE) after Year 12, and are randomly assigned into classes stratified by gender and NHEEE score at the time of the first enrolment. All third year students studying clinical medicine in the eight-year training program were informed about the study at one of their routine administrative meetings and recruited by an administrative staff member from the medical school. At the time of participation, they had completed only basic science subjects. The four classes were randomly assigned to each intervention by a blinded administrator.

Written informed consent was obtained from all participants after full explanation of the study. The study was approved by the University of Sydney Human Research Ethics Committee and the Peking University Health Science Centre Human Research Ethics Committee.

### Intervention

The educational intervention package was designed with the aim of combining evidence-based educational intervention strategies, with consideration of the teaching and administrative environment for medical students in China. The timing of the study was selected to fit into "Promotion and Education Month" which occurs during the first month of each semester at Peking University Medical Science Centre. The aim is improving knowledge and understanding of a particular health condition. The theme of "Better Understanding of Depression" was given in the particular month when the study was conducted.

The four classes were assigned into two groups: the didactic teaching group (DT) and the didactic teaching and self-directed learning group (DT/SDL). Both groups together had a standard 1.5 hour lecture which covered all the basic medical aspects about depression required by the teaching guidelines, including incidence and prevalence (in the world and in China), social and economic impacts (the world and China), common psychological and physical symptoms, case examples, treatments and prognosis of depression.

Immediately after the lecture, the DT/SDL group students were divided into six study groups within the classes and completed the following activities over the following 10 days under assistance from a researcher (YR) and school administrators:

1) All students were asked to search for information on various aspects about depression and to develop an understanding of the importance of depression to individuals and society. Each study group was required to design and organise a half-day advocacy activity about depression in a public place by setting up a display board. They were encouraged to talk with people about depression and understand public perceptions of depression.

2) After this activity, the students attended a 1.5-hour group session. In each group, the session started with a student-centred activity using a creative or artistic method to express their understanding of people's life with depression (e.g. role play, talk show, song or dance). The use of the arts in medical education and training has been reported to improve communication, empathy and understanding of patients' needs [20]. Then, they watched an 18-minute long video on depression (including the lived experience of a student with depression, a celebrity's talk on his depression and an expert commenting on the condition), followed by a discussion focusing on depression and its impact on people's life. In total, the amount of time students spent on these activities in the DT/SDL group was estimated as 20 hours over 10 days.

### Measures

The knowledge of and attitudes towards depression were assessed by using the same self-report questionnaire immediately prior to the lecture (baseline), two weeks after baseline (first follow-up, FU1), and one month and six months after the intervention for both groups (second and third follow-ups, FU2 and FU3).

Knowledge of depression was assessed using questions from the International Depression Literacy Survey (IDLS). The IDLS was developed to investigate the knowledge about general and mental health issues, as well as attitudes and personal mental health experience. It consists of individual perceptions of major health and mental health problems in their countries, knowledge regarding the typical symptoms and common experience of depression and opinion on treatment and recovery. The utility of IDLS has been demonstrated among medical and non-medical students in both Australia and China [8,21]. In terms of face and construct validity, it was able to detect clear differences between medical students in second and fourth years courses, and between non-medical students from ethnic Chinese backgrounds and other undergraduates residing in Australia [22]. The level of knowledge and recognition of depression was assessed in three ways: the proportion of students nominating depression as a main cause of death or disability in China (public health impact), the proportion of students nominating specific common behaviours or experiences for a person with depression (recognition) and the proportion indicating that recovery was possible and that antidepressants would be useful (outcome).

The students' attitudes to depression were assessed using the Mental Illness: Clinician's Attitude's (MICA) scale which was specially designed for assessing the level of stigmatising attitudes to mental illness and psychiatry among medical students [23]. The MICA scale has satisfactory internal consistency, face and construct validity. It includes 16 items. Each item is rated by using a six-point Likert scale from 1 to 6 indicating 'strongly agree', 'agree', 'somewhat agree', 'somewhat disagree', 'disagree', and 'strongly disagree', respectively. The MICA was adapted to this study with modification of the phrase "mental illness" being translated as "depression".

Both the IDLS and the MICA were forward and back translated into Chinese (Mandarin) with a face validation for semantic consistency with bilingual health professionals. Information on demographics (age, gender and area of origin), personal and social experience with depression, and current psychological distress status (K10 which measures psychological symptoms on a 10-50 scale) were also collected at baseline as being previously embedded in the IDLS [22].

### Data analysis

Descriptive statistics (means, numbers and proportions) were performed for the demographic data. At baseline, comparisons of the two groups were assessed. Chi-squared tests were used to test for associations between categorical variables and group. All continuous variables were examined for linearity and distribution. T-tests were performed for these associations.

The change in knowledge about depression was evaluated by comparing the proportions of students among baseline and the follow-ups using a series of Generalised Estimating Equations (GEE). As there was a specification of the number of responses within each of these knowledge questions, only students nominating a certain number of responses were included in the analyses for the item. To assess the impact of the interventions on attitudes towards depression, we conducted further analyses using GEE examining the mean MICA scores (greater mean of MICA scores implying more stigmatising attitudes towards depression).

In each of the GEE analyses, group (DT vs. DT/SDL) and time (baseline vs. FU1 vs. FU2 vs. FU3) were fixed factors, and the procedure tested for main effect (time) and group by time interaction effect. In addition, repeated contrasts were run, and the comparison between baseline and each subsequent time point was examined within each group separately. All analyses were adjusted for baseline values (e.g. baseline MICA score was a covariate for all the MICA comparisons). *P *was set at 0.05 for all analyses.

The effect sizes were measured by the odds ratio of the proportion of students nominating depression as a main cause of death or disability and the standardised difference of means of MICA score between the groups at FU1.

## RESULTS

### Demographic characteristics

There were 205 medical students who participated in the study: 103 students in the DT group and 102 in the DT/SDL group. There were no significant differences in the demographic characteristics of participants between the groups at baseline (Table 1). There was no significant difference in psychological distress, as measured by the K10, between the groups (16.42 vs. 16.69, *t *= 0.43, d.f. = 198, *P *= 0.668). There were no significant differences in depression knowledge or attitudes between the two groups at baseline (proportion of students nominating depression as a main cause of death or disability: X^2 ^= 0.30, d.f. = 1, *P *= 0.582; MICA score 43.75 vs. 43.27, *t *= 0.51, d.f. = 203, *P *= 0.613).

**Table 1:** Characteristics of the DT group and the DT/SDL group at baseline

		Total	DT	DT/SDL	Statistical comparison of DT vs DT/SDL
					
		N = 205	**n**_**1 **_**= 103**	**n**_**2 **_**= 102**	
Age, years: mean (SD)		20.18 (0.70)	20.23 (0.70)	20.13 (0.70)	*t *= 1.00, d.f. = 202, *P *= 0.319

Gender, *n *(%)					
	Male	91 (44.4)	46 (44.7)	45 (44.1)	X^2 ^= 0.01, d.f. = 1, *P = *0.938
	Female	114 (55.6)	57 (55.3)	57 (55.9)	

Area of origin, *n *(%)					
	Urban	121 (59.0)	65 (63.1)	56 (54.9)	X^2 ^= 1.43, d.f. = 1, *P *= 0.232
	Non-urban	84 (41.0)	38 (36.9)	46 (45.1)	

Experience depression, *n *(%)					
	Yes	28 (86.3)	14 (13.6)	14 (13.7)	X^2 ^= 0.00, d.f. = 1, *P = *0.978
	No	177 (13.7)	89 (86.4)	88 (86.3)	

Depression nominated as a main cause of death or disability, n (%) ^b^					
	Yes	57 (35.0)	30 (37.0)	27 (32.9)	X^2 ^= 0.30, d.f. = 1, *P *= 0.582
	No	106 (65.0)	51 (63.0)	55 (67.1)	

MICA score: mean (SD)		43.51 (6.67)	43.75 (6.93)	43.27 (6.42)	*t *= 0.51, d.f. = 203, *P *= 0.613

Psychological distress (K10) ^a^: mean (SD)		16.55 (4.46)	16.42 (4.20)	16.69 (4.72)	*t *= 0.43, d.f. = 198, *P *= 0.668

^a ^The respondent scores of 21 or below indicate low or moderate level of psychological distress; the respondent scores of 22 or above indicate high or very high level of psychological distress.

^b ^Only those students who nominated over four diseases at baseline were included in this analysis.

### Knowledge

#### 1) Public health impact

Students were requested to choose up to six main causes of death or disability from a list of specific illnesses or injuries. There were 191 students who answered the question correctly (choosing not more than six items) at each time point. The average numbers of nomination were 4.68 (SD 1.31) at baseline, 4.47 (SD 1.41) at FU1, 4.63 (SD 1.32) at FU2 and 4.40 (SD 1.37) at FU3. Among the 191 medical students, only the 95 (49.7%) students who nominated four or more illnesses or injuries throughout the entire study are included in this analysis to enable comparisons. There was a very small difference in age (20.09 vs. 20.28, *t *= 0.17, d.f. = 185, *P *= 0.048), but no difference in proportions of male students and students from urban origin between the students who nominated four or more illness or injuries at each time point and those who did not. The proportions of students nominating each of the top six illness or injuries as a main cause of death or disability at baseline and each follow-up time point are depicted in Table 2.

**Table 2:** Proportion of students nominating specific illnesses or injuries as a main cause of death or disability (N = 95)*

		**DT (n**_**1 **_**= 46)**				**DT/SDL (n**_**2 **_**= 49)**			
		
		Baseline	FU1	FU2	FU3	Baseline	FU1	FU2	FU3
		*n *(%)	*n *(%)	*n *(%)	*n *(%)	*n *(%)	*n *(%)	*n *(%)	*n *(%)
1	Heart attack or other heart diseases	32 (69.6)	25 (54.3) ^a^	28 (60.9)	36 (78.3)	34 (69.4)	29 (59.2)	37 (75.5)	34 (69.4)
2	HIV infection or AIDS	29 (63.0)	25 (54.3)	21 (45.7) ^a^	25 (54.3)	30 (61.2)	29 (59.2)	25 (51.0)	18 (36.7) ^c^
3	Diabetes	28 (60.9)	22 (47.8)	20 (43.5) ^a^	23 (50.0)	29 (59.2)	31 (63.3)	28 (57.1)	23 (46.9)
4	Road traffic accidents	30 (65.2)	26 (56.5)	22 (47.8)	22 (47.8)	27 (55.1)	29 (59.2)	25 (51.0)	21 (42.9)
5	Stroke or other brain disease	20 (43.5)	19 (41.3)	18 (39.1)	20 (43.5)	29 (59.2)	16 (32.7) ^c^	24 (49.0)	24 (49.0)
6	Depression	17 (37.0)	22 (47.8)	22 (47.8)	26 (56.5) ^e^	19 (38.8)	35 (71.4) ^b^	35 (71.4) ^b^	31 (63.3) ^d^

* Only the students who nominated at least four illnesses or injuries at each time point were included in this analysis. These were the most common illnesses or injuries nominated by students as a main cause of death or disability at baseline.

^a ^*P *< 0.05 compared with baseline

^b ^*P *< 0.001 compared with baseline

^c ^*P *= 0.001 compared with baseline

^d ^*P *< 0.005 compared with baseline

^e ^*P *= 0.051, the pairwise comparison between baseline and FU3 for the DT group indicated a trend towards a difference in proportion of depression nomination.

Only 36 of the 95 students (37.9%) nominated depression as a main cause of death or disability at baseline. This proportion did not vary by age, gender, area of origin, personal experience of depression or the level of psychological distress. After the intervention, regarding the changes in the proportions of students nominating "*depression" *as a main cause of death or disability, there was a significant time effect and a group by time interaction effect (Wald X^2 ^= 18.75, d.f. = 3, *P *< 0.001; Wald X^2 ^= 25.89, d.f. = 7, *P *= 0.001; respectively), indicating a significant overall increase in the proportion of students nominating "*depression*" across time and a significantly larger increase in the DT/SDL group over time. Specific contrasts between baseline and each subsequent time point, presented in Table 2, reflect this group by time effect, with the DT/SDL group having a significantly higher proportion of students nominating depression at each post-intervention follow-up, whereas the DT group only differed from baseline at FU3. This suggests that the preferential effect of the intervention upon knowledge of the public health impact of depression had waned by six months, partly through increase in knowledge in the control group.

Of note there were no significant time effects or group by time interaction effects for "*diabetes*", "*road traffic accidents*" or "*stroke or other brain disease*". For "*heart attack or other heart disease*" and "*HIV infection or AIDS*', there were significant time effects and group by time interaction effects. These reflected a significant overall decrease in the proportion of students nominating "*heart attack or other heart disease*" and "*HIV infection or AIDS*' as a main cause of death or disability (Wald X^2 ^= 11.16, d.f. = 3, *P *= 0.011; Wald X^2 ^= 11.79, d.f. = 3, *P *= 0.008; respectively), as the proportion of students nominating "*depression*" rose, and significant differences in the change of the proportions between the two groups across time (Wald X^2 ^= 15.90, d.f. = 7, *P *= 0.026; Wald X^2 ^= 18.10, d.f. = 7, *P *= 0.012; respectively), effects that disappeared at six months.

#### 2) Recognition: typical symptoms, signs and behaviours of depression

Students were asked to nominate up to five typical signs or symptoms for a person with depression. There were 170 students who answered the question correctly (choosing not more than five items) at each time point. The average numbers of signs nominated were 4.33 (SD 0.90) at baseline, 4.43 (SD 0.90) at FU1, 4.38 (SD 0.90) at FU2 and 4.32 (SD 0.89) at FU3. Among these 170 students, there were 146 (85.9%) students who nominated at least three typical signs or symptoms throughout the study, and they were included in the analysis. There was no difference in age or proportions of male students and students from urban origin between the students who nominated three or more typical signs or symptoms at each time point and those who did not. The top five typical signs or symptoms for a person with depression as nominated by these students are reported in Table 3. Whilst the proportions of the students nominating each symptom as typical for a person with depression fluctuated over time, the five symptoms of *"feeling sad, down, or miserable*", "*sleep disturbance*", "*being unhappy or depressed*", "*feeling overwhelmed*", and " *thinking 'life is not worth living'*, remained the most commonly nominated symptoms throughout the study.

**Table 3:** Proportion of students nominating typical signs or symptoms for a person with depression (N = 146)*

		**DT (n**_**1 **_**= 69)**				**DT/SDL (n**_**2 **_**= 77)**			
		
		Baseline	FU1	FU2	FU3	Baseline	FU1	FU2	FU3
		*n *(%)	*n *(%)	*n *(%)	*n *(%)	*n *(%)	*n *(%)	*n *(%)	*n *(%)
1	Feel sad, down, or miserable	39 (56.5)	41 (59.4)	34 (49.3)	40 (58.0)	47 (61.0)	53 (68.8)	53 (68.8)	49 (63.6)
2	Sleep disturbance	34 (49.3)	25 (36.2) ^a^	26 (37.7)	17 (24.6) ^b^	37 (48.1)	41 (53.2)	28 (36.4) ^a^	29 (37.7)
3	Unhappy or depressed	29 (42.0)	34 (49.3)	34 (49.3)	40 (58.0) ^a^	39 (50.6)	37 (48.1)	34 (44.2)	40 (51.9)
4	Overwhelmed	31 (44.9)	18 (26.1)	24 (34.8) ^a^	20 (29.0)	33 (42.9)	33 (42.9)	26 (33.8)	31 (40.3)
5	Thinking "life is not worth living"	23 (33.3)	27 (39.1)	16 (23.2)	28 (40.6)	22 (28.6)	32 (41.6)	33 (42.9) ^a^	28 (36.4)

* Only the students who nominated at least three typical signs or symptoms at each time point were included in this analysis. These were the top five typical signs or symptoms for a person with depression that were nominated by students at baseline.

^a ^*P *< 0.05 compared with baseline

^b ^*P *< 0.001 compared with baseline

Students were asked to nominate from a list up to four common behaviours or experiences for a person with depression. There were 179 students who answered the question correctly (choosing not more than four items) at each time point. The average nominations were 3.27 (SD 0.94) at baseline, 3.42 (SD 0.84) at FU1, 3.45 (SD 0.79) at FU2 and 3.35 (SD 0.80) at FU3. Among these 179 students, only the 113 (63.1%) students who nominated three or four common behaviours or experiences throughout the study were included in this analysis (Table 4). There was no difference in age or proportion of students from urban origin, but a difference in proportion of male students (44/113 (38.9%) vs. 37/66 (56.1%), *P *= 0.039) between the students who nominated three or four common behaviours or experiences at each time point and those who did not.

**Table 4:** Proportion of students nominating common behaviours or experiences for a person with depression (N = 113) *

		DT (n = 48)				DT and SDL (n = 65)			
		
		Baseline	FU1	FU2	FU3	Baseline	FU1	FU2	FU3
		*n *(%)	*n *(%)	*n *(%)	*n *(%)	*n *(%)	*n *(%)	*n *(%)	*n *(%)
1	Suicidal thoughts or behaviour	37 (77.1)	27 (56.2) ^c^	26 (54.2) ^b^	26 (54.2) ^c^	38 (58.5)	39 (60.0)	30 (46.2)	38 (58.5)
2	Having relationship or family problem	31 (64.6)	29 (60.4)	29 (60.4)	31 (64.6)	44 (67.7)	40 (61.5)	43 (66.2)	48 (73.8)
3	Cannot concentrate or have difficulty thinking	19 (39.6)	26 (54.2)	34 (70.8) ^d^	20 (41.7)	34 (52.3)	46 (70.8) ^a^	47 (72.3) ^a^	47 (72.3) ^a^
4	Stop going out	13 (27.1)	10 (20.8)	10 (20.8)	10 (20.8)	18 (27.7)	15 (23.1)	9 (13.8) ^a^	5 (7.7) ^c^
5	Withdraw from close family and friends	19 (39.6)	15 (31.2)	12 (25.0)	20 (41.7)	15 (23.1)	12 (18.5)	6 (9.2) ^a^	7 (10.8)

* Only the students who nominated three or four common behaviours or experiences at each time point were included in this analysis. These were the top five common behaviours or experiences for a person with depression that were nominated by students at baseline.

^a ^*P *< 0.05 compared with baseline

^b ^*P *< 0.01 compared with baseline

^c ^*P *< 0.005 compared with baseline

^d ^*P *< 0.001 compared with baseline

There were significant group by time interaction effects for "*suicidal thoughts or behaviour*" (Wald X^2 ^= 19.54, d.f. = 7, *P *= 0.007) and "*be unable to concentrate or have difficulty thinking*" (Wald X^2 ^= 33.59, d.f. = 7, *P *< 0.001). While the proportion of students nominating "*suicidal thoughts or behaviour*" as a common behaviour of depression remained relatively steady in the DT/SDL group, there was a significant decrease in the DT group, as demonstrated by the contrasts between the baseline and each follow-up time point. In the DT/SDL group, the proportion of students nominating "*be unable to concentrate or have difficulty thinking*" increased significantly, as indicated by the contrasts between the baseline and each follow-up time point, while there was no significant change in the DT group. There were also significant group by time interaction effects for "*stop going out*" (Wald X^2 ^= 20.83, d.f. = 7, *P *= 0.004) and "*withdraw from close family and friends*" (Wald X^2 ^= 35.26, d.f. = 7, *P *< 0.001). In the DT/SDL group, the decrease in the proportion of the students nominating "*stop going out*" progressed and was significant according to the contrasts between the baseline and the FU2 and FU3 time points; the proportion of students nominating "*withdraw from close family and friends*" also decreased across time, but only the contrast between baseline and FU2 was significant.

#### 3) Treatment and Outcome

**Figure 1:**
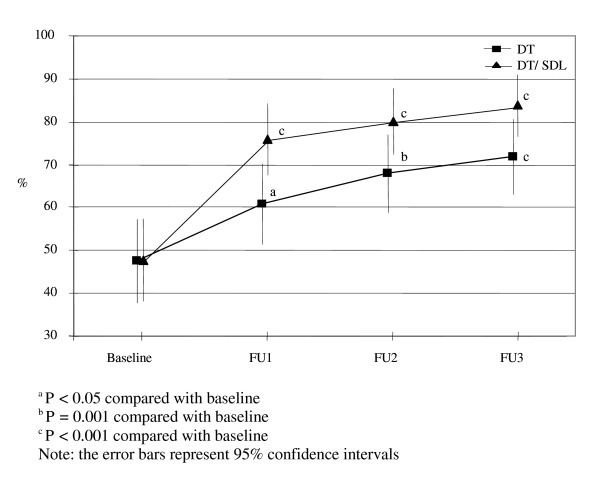
**Proportion of students believing antidepressant as a helpful treatment for depression**.

There were significant time and group by time interaction effects for the proportion of students who considered "*antidepressant medications*" as a helpful treatment for depression (Wald X^2 ^= 75.62, d.f. = 3, *P *< 0.001; Wald X^2 ^= 87.76, d.f. = 7, *P *< 0.001; respectively), indicating an overall increase in the proportion of students considering "*antidepressant medications*" as helpful in both groups across time, but a significantly larger increase in the DT/SDL group compared with the DT group (Figure 1).

**Figure 2:**
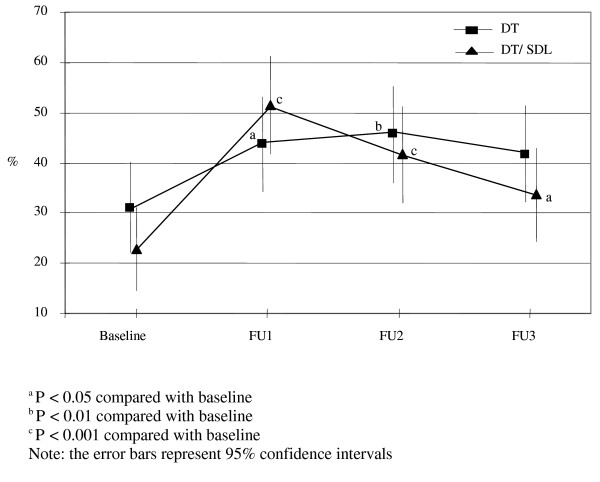
**Proportion of students believing a possible full recovery of depression with professional help**.

Similarly there were significant time and group by time interaction effects for the proportion of students who considered full recovery from depression was likely with professional help (Wald X^2 ^= 28.23, d.f. = 3, *P *< 0.001; Wald X^2 ^= 34.76, d.f. = 7, *P *< 0.001; respectively). Again the increase was considerably larger between baseline and FU1 (immediately after the intervention) in the DT/SDL group (Figure 2).

### Attitudes towards depression

At baseline, there was a significant difference in attitudes, assessed using the MICA scale, between the female and male students. Male students had higher scores reflecting more stigmatising attitudes compared with the female students (44.58, SD 7.07 vs. 42.66, SD 6.23, *t *= 2.07, d.f. = 203, *P *= 0.040). There was no significant association between attitude scores and age, area of origin, experience with depression and personal level of psychological distress.

**Figure 3:**
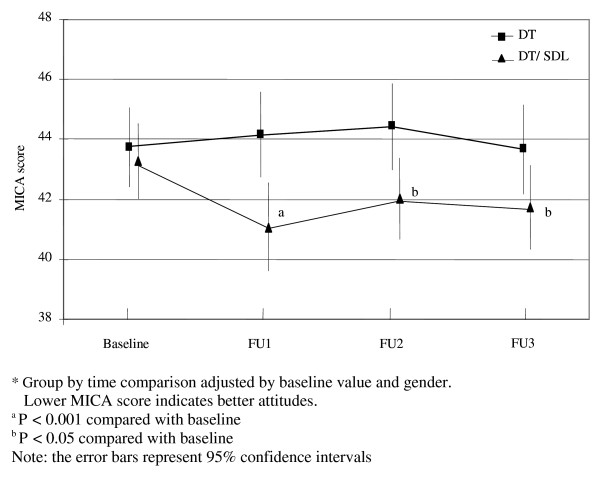
MICA score*.

There was a significant group by time interaction effect of the intervention on the MICA scores (Wald X^2 ^= 19.45, d.f. = 7, *P *= 0.007) after adjusting for baseline MICA score and gender. In the DT group, the MICA scores at all follow-up time points were comparable to the baseline MICA score (Baseline: 43.75, SD 0.68; FU1: 44.15, SD 0.73; FU2: 44.43, SD 0.73; and FU3: 43.67, SD 0.77). However, in the DT/SDL group, the MICA scores decreased and remained lower, as demonstrated by the contrasts between baseline (43.27, SD 0.63) and each follow-up time point (FU1: 41.10, SD 0.74, *P *< 0.001; FU2: 42.04, SD 0.68, *P *= 0.033; FU3: 41.73, SD 0.70, *P *= 0.011 ) (Figure 3).

### Effect size

As the measure of effect size on recognition of public health importance of depression, the odds ratio of the proportion of students nominating depression as a main cause of death or disability between the DT/SDL group and the DT group was 2.88 (95% CI: 1.48 - 5.59) at FU1. The standardised difference of response means, as a measure of effect size on attitudes towards depression, was 0.42 between the DT/SDL group and the DT group for the MICA score at FU1. This is a small to moderate effect using Cohen's guidelines [24].

## Discussion

This study suggests that the combined didactic teaching and self-directed learning strategy employed in an anti-stigma education for depression among Chinese medical students resulted in an improvement in knowledge of public health and treatment aspects of depression and a sustained reduction in stigmatising attitudes towards depression. By contrast, the traditional didactic lecture only moderately improved the knowledge of depression and had no effect upon attitudes. The dramatic increase in recognition of depression as a main cause of death or disability in the DT/SDL group (despite information on this being in the lecture received by all students) indicated that the self-directed learning intervention was more effective in improving the understanding of the public health impact of depression, although the differential effect had waned after six months of training in other medical specialties.

While there was little differential effect of the intervention on many aspects of clinical knowledge of depression in terms of typical symptoms and common behaviours, the impact of the self-directed learning intervention on the confidence of the helpfulness of antidepressant medication and the recovery of depression should be acknowledged. All of these were highlighted in the didactic lecture but the self-directed learning seemed to embed more knowledge than just basic clinical signs and symptoms which many lay people could list. Active detection and management of a mental disorder by a physician is associated with a strong sense of urgency, a high level of certainty, and positive self-perception and attitudes [25,26]. The results from this study demonstrated that the self-directed leaning strategy was effective in improving the confidence of treatment and outcome for depression, and could be promoted in education among health professionals for proactive diagnosis and treatment of depression.

One important aspect of this study was the finding of persistent changes following the intervention, particularly in attitudes. Previous research in medical students has demonstrated that the short term effect of more didactic interventions decays rapidly [17]. Other studies have shown significant and moderately sustained effects of an intensive 12-hour course on improving knowledge, attitudes and helping behaviours and reducing social distance in community subjects [27], while an interactive web-based intervention had a strong effect on improving knowledge and reducing stigma amongst students [28]. However, long term availability of quality information and frequent mass media exposure through specific public health campaigns have also been shown to improve knowledge of and attitudes towards depression by building up a supportive and information-filled environment, rather than using concentrated educational sessions [29]. It is unclear whether the effect in our study was a result of the more intensive intervention, the student-centred teaching style or the open and depression-supportive teaching environment.

In terms of implementation, the specific educational culture underpinned the provision of intervention in this study. The school administration plays a central role in management of the study activities and campus life of the medical students. While they take care of over 200 students, there is very limited time for extra commitments. The design of the study was based on their existing responsibility, work pattern and schedule. The staff members were highly enthusiastic about the innovative project due to an increased awareness of mental disorders and suicide among college students in recent years. The education activities designed in this study not only accorded with the university's new education principle, but encouraged students' involvement, creativity and team work. In addition, the project was conducted at the beginning of a semester when the medical students were less busy in their study.

Some limitations of this study are notable. First, the clinical knowledge items on depression in IDLS (symptoms and experiences items) limited the number of responses. For any particular item, a decrease in the proportion of students nominating it may have resulted from an increase in the proportion of students nominating another and may not reflect any real pattern of change in clinical knowledge. For example, in the DT/SDL group, while the proportion of students nominating "*unhappy or depressed*" as a typical symptom for people with depression increased from 34 (44.2%) at FU2 to 40 (51.9%) at FU3, the proportion of students nominating "*feel sad, down or miserable*" decreased from 53 (68.8%) at FU2 to 49 (63.6%) at FU3. Second, the MICA scale was originally developed in English for assessment of attitudes towards "mental illness" in general. A few items were modified to assess attitudes towards depression in particular before the scale was translated into Chinese (translation and back translation). Third, groups of students, rather than individuals were randomised to receive the intervention. However, the students had been randomly assigned into the classes when they entered the university and had comparable study and life environments. As the students could not be blinded to the intervention due to the expectation of understanding the tasks and the need to follow instructions, the school administrator was alternatively blinded to the allocation of the interventions. We suspect there was very little contamination of intervention because students from different classes stayed in separated dormitories and had different timetables. Finally, self-reported questionnaires are open to specific response biases. Those students exposed to the intervention might be expected to give responses that were consistent with the goals and content of the depression-related activities.

Other considerations include recognising that this intervention may be difficult to deliver in other Chinese and non-Chinese educational institutions. The unique cultural nature of education in Peking University may not be comparable with other sites. The intervention was conducted among third-year undergraduate medical students, thus the results may not be generalised to different years of medical study or students in non-undergraduate medical training. In addition, while successful for a common mental health problem like depression, the intervention may not be so powerful for other mental disorders such as schizophrenia.

## Conclusions

The World Health Organisation (WHO) recommended a comprehensive curriculum in psychiatry, in a student-centred method, to prepare medical students with adequate knowledge, skills and attitudes in non-psychiatric care [30]. As Chinese health service priorities change to cope with more chronic disease including mental illness in a primary health care setting, the country needs corresponding training strategies for health professionals. This study suggests a context-specific, student-centred intervention of relatively high intensity can produce durable knowledge and attitudinal changes. Whether this translates into later enhancements in practitioner behaviour or direct benefits to patients and carers is the subject of ongoing work.

## Competing interests

The authors declare that they have no competing interests.

## Authors' contributions

YR designed the study, analysed the data and drafted the manuscript. NG and GML participated in the statistical analysis and drafting the manuscript. TAD participated in the study design and drafting the manuscript. YH participated in carrying out the study and interpreting the data. IBH participated in the study design and critically appraised the manuscript. All authors read and approved the final manuscript.

## Pre-publication history

The pre-publication history for this paper can be accessed here:

http://www.biomedcentral.com/1471-244X/11/36/prepub
